# Pentosan polysulfate alleviates interstitial cystitis/bladder pain syndrome by modulating bile acid metabolism and activating the TGR5 receptor through gut microbiota regulation

**DOI:** 10.14440/bladder.2024.0060

**Published:** 2025-03-24

**Authors:** Zhangrui Zhu, Yuexuan Zhu, Qi Sun, Jingwen Xue, Ming Xie, Yao Yu, Benlin Wang, Wentai Shangguan, Zhengyuan Feng, Peng Wu

**Affiliations:** Department of Urology, Nanfang Hospital, Southern Medical University, Guangzhou, Guangdong 510515, China

**Keywords:** Interstitial cystitis/bladder pain syndrome, Pentosan polysulfate, Cyclophosphamide, Ursodeoxycholic acid, Bladder barrier, Gut microbiota

## Abstract

**Background:**

The disrupted gut microbiome has been found to be implicated in the development of interstitial cystitis/bladder pain syndrome (IC/BPS). Pentosan polysulfate (PPS) is an oral medication used for treating IC/BPS, acting as both an anti-inflammatory agent and a bladder barrier protector. However, the precise mechanisms by which the PPS-mediated modulation of the gut microbiome alleviates IC/BPS are not fully understood.

**Objective:**

This study aimed to identify the key gut microbiota species and metabolites involved in PPS’s protective effects against IC/BPS.

**Methods:**

We employed a multifaceted approach, including 16S rDNA gene sequencing, antibiotic treatment, and fecal microbiota transplantation, to validate the dependency of PPS’s protective effects on the gut microbiome. Furthermore, we performed a comprehensive metabolomic profiling using non-targeted metabolomics and liquid chromatography-tandem mass spectrometry.

**Results:**

PPS significantly elevated the abundance of the xylan-degrading bacteria, *Eubacterium xylanophilum* group, which, through its interaction with the gut microbiome, markedly reduced inflammation and barrier damage induced by cyclophosphamide in IC/BPS. In addition, PPS significantly increased the level of ursodeoxycholic acid (UDCA), a secondary bile acid, demonstrating a strong correlation with the abundance of the *E. xylanophilum* group. *Ex vivo* supplementation with UDCA mitigated lipopolysaccharide-induced inflammation and barrier disruption in SV-HUC-1 cells by activating the TGR5 receptor.

**Conclusion:**

PPS exerts its protective effects against IC/BPS by modulating the gut microbiome and its metabolites.

## 1. Introduction

Interstitial cystitis/bladder pain syndrome (IC/BPS) is characterized by chronic bladder discomfort and lower urinary tract symptoms persisting for over 6 weeks without a clear infection or other identifiable causes. Women are 2 – 5 times more likely to develop IC/BPS than men, with a prevalence of up to 14.8% in women over 25.^1^,^2^ Despite ongoing research, the exact etiology of IC/BPS remains unclear. However, increased inflammatory factors and damage to the bladder epithelial barrier can lead to the infiltration of toxic substances (such as potassium ions in urine) into submucosal nerves, ultimately resulting in the characteristic clinical symptoms of IC/BPS.^3^ The urothelium, a specialized epithelial lining of the bladder, plays a crucial role in maintaining the bladder’s integrity and functional barrier. Changes in urothelial function are intimately related to the pathophysiological mechanisms of IC/BPS, regardless of the presence or absence of Hunner’s lesions.^4^ In fact, Hunner’s lesions, ulcer-like areas on the bladder wall, are a hallmark of certain types of IC/BPS. Understanding the intricate interactions among urothelial dysfunction, inflammation, and barrier damage is crucial for developing efficacious therapies for IC/BPS.

The gut microbiota, a complex community of microorganisms residing in the gastrointestinal tract, plays a fundamental role in shaping the host’s innate and adaptive immune responses.^5^ Besides its immunoregulatory functions, the gut microbiota profoundly influences host physiology by regulating intestinal motility, maintaining intestinal barrier integrity, and influencing various metabolic processes.^6^ Mounting evidence suggests that the influence of the gut microbiota may extend beyond the gastrointestinal tract, to potentially impact intestinal and bladder function by effecting metabolic or neuromodulatory changes. This has led to the concept of a microbiota-mediated gut-bladder axis, which has been implicated in the pathophysiology of a wide array of disorders, including irritable bowel syndrome (IBS),^7^ overactive bladder, IC/BPS, and chronic prostatitis/chronic pelvic pain syndrome.^8^ Patients with IC/BPS tend to have comorbidities, with about 50% reporting other conditions, primarily IBS.^9^ Regarding changes in the gut microbiota, a study showed a significantly lower abundance of certain gut bacteria in IC/BPS patients, including *Monascus* and lactate-producing bacteria than in healthy controls.^10^ These findings indicate that alterations in the gut microbiota may contribute to the pathophysiology of IC/BPS and its associated comorbidities, such as IBS. Understanding the complex relationship between the gut microbiota and IC/BPS and its potential role in the gut-bladder axis may help develop new therapeutic strategies for managing this debilitating disease and its related symptoms.

Pentosan polysulfate (PPS), derived from beechwood xylan, is the only Food and Drug Administration-approved oral treatment for IC/BPS, and can effectively alleviate symptoms such as frequency, urgency, and pain.^11^-^13^ However, long-term PPS use has been associated with retinal maculopathy, raising concerns about its safety.^14^ Despite these concerns, the potential of PPS as a prebiotic has garnered significant attention. When metabolized by gut microbiota, PPS produces xylan metabolites that are beneficial to gut health.^15^ In addition, PPS can affect major organs, such as liver, lungs, and brain through the gut-liver axis, gut-lung axis, and gut-brain axis, respectively, exerting its effects through systemic circulation.^16^-^18^ These findings suggest that PPS may have broader health benefits beyond its specific indication for IC/BPS.

In this study, we utilized cutting-edge techniques, including gut microbiota 16s rDNA sequencing, serum metabolomics, and liquid chromatography-mass spectrometry (LC/MS) to investigate the effects of PPS on the bladder urothelium and its role in managing IC/BPS through gut microbiota modulation. Our objectives were to identify key metabolites and molecular targets influenced by PPS and to gain insights into IC/BPS pathophysiology. This research also sought to discover alternative treatments to PPS for clinical application.

## 2. Materials and methods

### 2.1. Construction of cyclophosphamide (CYP)-induced IC/BPS model and drug intervention in mice

Female C57BL/6 mice (36), aged 6 – 8 weeks and weighing 18.2 ± 2.1 g, were used (SPF Biotechnology Co., Ltd., China) for the induction of IC/BPS. The mice were maintained under specific pathogen-free conditions with a controlled environment of 24 ± 1°C and 50 ± 10% relative humidity on a 12-h light/dark cycle, ensuring their wellbeing. They had *ad libitum* access to standard chow and water. Fecal samples were collected before euthanasia, and sterile blood and cecal content samples were obtained under anesthesia for subsequent analysis. Randomization was employed to assign mice to experimental and control groups to prevent selection bias. The randomization sequence was generated using a random number table, and the allocation was conducted by a researcher not involved in the subsequent experiments to ensure the integrity of the randomization process. All procedures were performed by trained personnel following standardized protocols to minimize variability. We set up the following experimental groups: control, PPS-only, CYP-only, and CYP + PPS groups. Mice were acclimated to a 12-h day/night cycle for 1 week before being reassigned to different cages to avoid cage bias. CYP and PPS were administered at 50 mg/kg and 25 mg/kg, respectively. CYP was injected intraperitoneally at a dose of approximately 0.1 mL per injection site, targeting the right lower abdominal triangle, after confirming the absence of significant blood or urine aspiration. PPS was given via intragastric gavage continuously for 3 weeks following a 7-day adaptive feeding period. The IC/BPS model was established with intraperitoneal injections of 50 mg/kg CYP on days 0, 3, and 5 of the 4^th^ week.^22^,^23^

This study was conducted in strict accordance with the ethical standards of the Institutional Animal Care and Use Committee (IACUC) of Southern Medical University, under protocol number IACUC-LAC-20221023-004. The IACUC ensures the humane treatment of animals and approved all experimental procedures involving animals, including housing conditions, anesthesia protocols, and methods of euthanasia.

### 2.2. Cell culture and treatment

SV-HUC-1 cells, immortalized human bladder epithelial cells from the New Laboratory of Southern Medical University, were cultivated in Ham’s F-12K medium supplemented with streptomycin (100 mg/mL) and penicillin (100 U/mL), along with 10% (v/v) fetal bovine serum, under conditions of 5% CO_2_ at 37°C. To model IC/BPS inflammation, the cells were treated with 50 μg/mL lipopolysaccharide (Aladdin Biochemical Technology Co., Ltd., China) for 24 h, following pre-treatment with 30 μM ursodeoxycholic acid (UDCA) (Aladdin Biochemical Technology Co., Ltd., China) for two h.^24^ For the TGR5 knockdown, shRNA knockdown was performed as previously described.^25^ The shRNA sequence was designed using the ThermoFisher online tool (https://rnaidesigner.thermofisher.com/) and obtained from Tsingke Biotechnology Co., Ltd., China. The knockdown sequences were:

(i). sh-TGR5F: CCGGGGTCTGGCATTGCCCACATTGC TCGAGCAATGTGGGCAATGCCAGACCTTTTTG
(ii). sh-TGR5-R: AATTCAAAAAGGTCTGGCATTGCCCA CATTGCTCGAGCAATGTGGGCAATGCCAGACC.

### 2.3. Antibiotic treatment and fecal microbiota transplantation (FMT)

To deplete the gut microbiota, female C57BL/6 mice were administered a cocktail of vancomycin hydrochloride (100 mg/kg), neomycin sulfate (200 mg/kg), ampicillin (200 mg/kg), and metronidazole (200 mg/kg) in normal saline (200 μL per mouse) through gavage for 5 days. For FMT, fecal samples were suspended in sterile phosphate-buffered saline (PBS) (0.125 mg/mL), vortexed, centrifuged, and mixed with 10% sterile glycerol. Mice received 200 μL of this suspension daily for 5 days following antibiotic treatment. Cecal contents from CYP and CYP + PPS mice were transplanted into antibiotic-treated mice to form the CYP-FMT and CYP + PPS-FMT groups, respectively, before exposure to CYP to induce IC/BPS.^26^,^27^

### 2.4. Fecal DNA extraction and 16S rDNA analysis

Fecal samples from mice were placed on ice and stored at −80°C for subsequent analysis. Microbial DNA was extracted from these samples as previously described.^28^,^29^ Bacterial DNA was amplified using polymerase chain reaction (PCR) with barcoded universal bacterial primers targeting the variable region V3-V4 of the 16S rDNA gene (341F, 5’-CCTACGGGNGGCWGCAG-3’; 806R, 5’-GGACTACHVGGGTATCTAAT-3’). Taxonomic analysis was performed using the Greengenes database. The raw sequencing reads were converted into clean tags by filtering low-quality reads, assembling sequences, and merging paired-end reads. These clean tags were then clustered, and chimeric tags detected during clustering were removed to produce effective tags. Operational taxonomic unit (OTU) abundance statistics were conducted based on these effective tags. α-diversity was assessed using the Chao1, Shannon, and Simpson indices, while β-diversity was analyzed using the principal coordinate analysis (PcoA) on the basis of the unweighted UniFrac distance. Diversity indices were calculated using the QIIME2 diversity plugin, and statistical significance between different groups was evaluated using permutational analysis of variance. Differential abundance analysis was performed using the LEfSe method to identify significantly different OTUs between groups, with a significance threshold set at a linear discriminant analysis score >2.0 and a *p* < 0.05. The 16S rDNA microbiome sequencing data were analyzed using the free online platform, Majorbio (https://www.majorbio.com/) (Majorbio Bio-Pharm Technology Co., Ltd., China).

### 2.5. Untargeted metabolomic analysis

Untargeted metabolomic profiling was conducted using LC-MS with the ultra-high-pressure liquid chromatography system (Agilent Technologies, USA). Data analysis was performed by Majorbio Bio-Pharm Technology Co., Ltd., China. The data were subjected to preprocessing, annotation, and multivariate statistical analysis, including the orthogonal partial least squares discriminant analysis (OPLS-DA) using the ropls R package for group comparisons. Metabolites with variable importance in projection (VIP) ≥ 1, *p* < 0.05, and an absolute fold change (FC) of ≥2 were identified as differentially expressed.

### 2.6. Measurement of serum UDCA

Serum samples (100 μL) were extracted using 1,400 μL acetonitrile, vortexed for 10 s, and centrifuged at 12,000 rpm for 15 min at 4°C. A 10 μL aliquot of the supernatant was injected into an XB-C18 column (Phenomenex, Cat# 00D-4496-AN, USA) and analyzed on an LC-MS system comprising a Shimadzu high-performance liquid chromatography system (Shimadzu Nexera LC-30A, Japan) coupled to an API 4000 triple quadrupole mass spectrometer (Applied Biosystems Sciex, Ontario, Canada). The mobile phases were water (A) and acetonitrile (B), with a gradient elution program as follows: 10% B for a minute, 95% B for 3.6 min, maintained at 95% B for 0.9 min, and then returned to 10% B for 2.1 min. Quantification was performed in multiple reaction monitoring modes for UDCA, with peak area integration by using the Thermo Scientific Xcalibur software (version 4.2.28.14 Thermo Fisher Scientific, USA).

### 2.7. Von Frey mechanical pain threshold detection in mice

The mice were placed in a box with a metal mesh floor and allowed to acclimate for 15 – 30 min (up to an hour if necessary) before testing. Von Frey filaments (starting from 0.008 g) were applied vertically to the bladder region of the lower abdomen until the fiber bent. Each stimulation lasted 3 – 5 s. A positive response was recorded (as “X”) if the mouse exhibited a rapid paw withdrawal or licking/scratching of the stimulated area within 3 – 5 s of stimulation. Adjacent smaller or larger fibers were used for retesting, with at least 10 s intervals between stimuli. If there were no responses, it was recorded as a negative response (“O”). A series of “O” or “X” combinations was obtained, with the “O” preceding the first “X” serving as the starting point. Six consecutive stimulus responses, including the starting point, such as “OXOXOO,” were selected to calculate the 50% mechanical pain threshold.^30^ Data were processed by utilizing the online up-down method for Von Frey experiments.^31^

### 2.8. Void spot assays (VSA)

**Figure S1:**
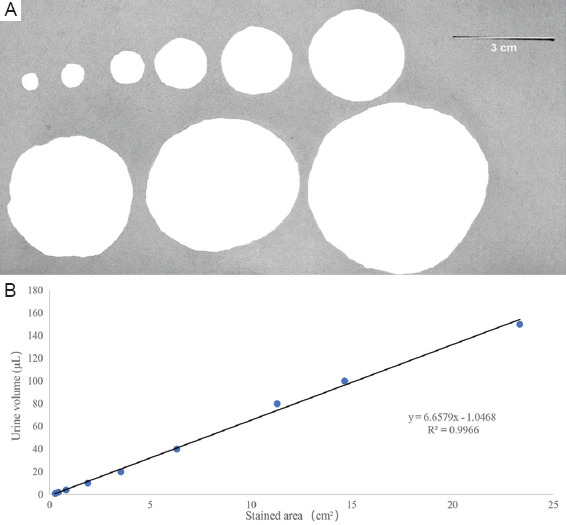
Standard curve of voiding spot on paper (VSOP). (A) Urine samples were collected to construct a standard curve and were dispensed onto filter paper in various volumes (1, 2, 4, 10, 20, 40, 80, 100, and 150 μL). (B) The formula y = 6.6579x-1.0468 (R² = 0.9966) was utilized to calculate individual void areas on the filter paper.

The VSA was employed to investigate the urinary frequency and mean voided volume.^32^ The self-constructed metabolic cage, a cubic metal structure measuring 18 × 17 × 30 cm, featured a wire mesh floor slightly smaller than the cage dimensions. A customized filter paper was inserted between the wire mesh and the cage bottom, allowing mice to move freely. At the same time, their urine onto the paper left visually detectable yellow spot-like prints. The cage minimized the impact of mouse feces, and activity traces on the filter paper, thereby preventing contamination of the urine prints and subsequent errors. During the experiment, mice were housed in the self-made metabolic cage with filter paper placed on the bottom and continuously monitored for 4 h. The filter papers were then collected and naturally air-dried. The Bio-Rad gel imaging analysis system (Bio-Rad Laboratories, United States), equipped with gel red for ultraviolet imaging, was used to capture images of the urine prints. These images were analyzed to determine urination frequency and average urination volume. Example plots and standard curves are shown in Figure S1.

### 2.9. Urodynamic measurements

Urodynamic measurements were conducted 24 h post-CYP administration in mice, following a previously described protocol.^33^ Mice were anesthetized with isoflurane (2%) and connected to a pressure transducer (Chengdu Taimeng Software Co., Ltd., China) and microinjection pump (Beijing Silugao Medical Technology Co., Ltd., China) via a T-tube. Before recording, the system was cleared of air. Normal saline was infused into the bladder at a rate of 1 mL/h to induce repetitive contractions. Continuous urodynamic curves were recorded for at least 30 min using the BL-420N biosignal analysis software (version 4.2.28.14, Chengdu Taimeng Software Co., Ltd., China). Micturition pressure and intercontractile intervals were analyzed to assess bladder contractility and urinary frequency.

### 2.10. Bladder histology

Mouse bladders were harvested and processed according to standard procedures, including fixation with 4% paraformaldehyde, paraffin-embedding, sectioning at 5 μm, dewaxing, hematoxylin and eosin (HE) staining, dehydration, and photography.

### 2.11. Glycosaminoglycan (GAG) detection

To quantify GAG levels in the bladder, an enzyme-linked immunosorbent assay (ELISA) was performed. Bladder samples were homogenized in PBS to obtain 10% tissue homogenates, and supernatants were collected using centrifugation at 12,000×g for 10 min. GAG concentrations were calculated using a commercial ELISA kit (Jiangsu Meimian Industrial Co., Ltd., China) by following the manufacturer’s instructions.

### 2.12. CCK8 cell viability assay

*In vitro* CCK8 cell viability assays were conducted using the CCK8 Kit (GlpBio, Cat# GK10001, USA), in accordance with the manufacturer’s protocol.

### 2.13. Quantitative real-time PCR

Total RNA was extracted from bladder samples using the Animal Total RNA Isolation Kit (Foregene Co., Ltd, China) and quantified using NanoDrop (BioTeke, China). Complementary DNA was synthesized from RNA using HiScript III RT SuperMix for quantitative PCR (qPCR) (Vazyme Biotech Co., Ltd, China), following the manufacturer’s guidelines. Gene expression levels of proinflammatory cytokines, tight junction proteins, and bile acid receptors were quantified using ChamQ SYBR qPCR Master Mix (Vazyme, China) on a LightCycler-^®^ 480 II Instrument, employing the 2^-ΔΔCt^ method.^34^ β-actin or *GAPDH* mRNA expression was used as a reference, with the control group serving as the baseline. The primary sequences used for qPCR are listed in Tables S1 and S2.

**Table S1:** The primary sequences of primers for quantitative PCR in mouse

Mus-gene	Forward primer (5’ – 3’)	Reverse primer (5’ – 3’)
β-actin	AGAGCTACGAGCTGCCTGAC	AGCACTGTGTTGGCGTACAG
*Tnfa*	AGGGTCTGGGCCATAGAACT	CCACCACGCTCTTCTGTCTAC
*Ilb*	CAGGCAGGCAGTATCACTCA	AGCTCATATGGGTCCGACAG
*Il6*	GAGCCCACCAAGAACGATAG	TCCACGATTTCCCAGAGAAC
*Zo1*	AGAGACAAGATGTCCGCCAG	TGCAATTCCAAATCCAAACC
Claudin-1	GCCATCTACGAGGGACTGTG	CCCCAGCAGGATGCCAATTA
Occludin	ACTCCTCCAATGGCAAAGTG	CCCCACCTGTCGTGTAGTCT

Abbreviation: PCR: Polymerase chain reaction

**Table S2:** The primary sequences of primers for quantitative PCR in cell

Homo- gene	Forward primer (5’ – 3’)	Reverse primer (5’ – 3’)
*GAPDH*	GAGTCAACGGATTTGGTCGT	TTGATTTTGGAGGGATCTCG
*TNFA*	TCCTTCAGACACCCTCAACC	AGGCCCCAGTTTGAATTCTT
*IL1B*	GCTGAGGAAGATGCTGGTTC	TCCATATCCTGTCCCTGGAG
*IL6*	AGGAGACTTGCCTGGTGAAA	CAGGGGTGGTTATTGCATCT
*ZO1*	TGAGGCAGCTCACATAATGC	GGTCTCTGCTGGCTTGTTTC
Claudin-1	GCCGTTGGCATGAAGTGTATG	GCCAGTGAAGAGAGCCTGAC
Occludin	CCTTCACCCCCATCTGACTA	GCAGGTGCTCTTTTTGAAGG
*GPBAR1*	CTGCCTCCTCGTCTACTTGG	GTAGGGGGCTGGGAAGATAG
*FXR*	ATCAAAGGGGATGAGCTGTG	CAGCCAACATTCCCATCTCT
*VDR*	GACGCCCACCATAAGACCTA	AGATTGGAGAAGCTGGACGA
*CAR*	TAATGCGCTGACTTGTGAGG	TCATGCCAGCATCTAAGCAC
*PXR*	CAAGGCTACGCTGACAATCA	CAGGGCTACATTTCCCAAAA
*GR*	ATAGCATGGGAGCTGGATTG	CCATGTGTTTTCATGGCTTG
*A5B1*	ACTCAAGCAAAAGGGAGCAA	TGCAAGCCTGTTGTATCAGC

Abbreviation: PCR: Polymerase chain reaction

### 2.14. Statistical analysis

Statistical analysis was conducted using GraphPad Prism software (version 9.0.0). Data are presented as mean ± standard error (mean±SEM). Comparisons between two groups were performed using Student’s *t*-test or Wilcoxon rank sum test, while comparisons between multiple groups were made using one-way analysis of variance. Significance levels were determined at *p* < 0.05 (*), *p* < 0.01 (**), *p* < 0.001 (***), and *p* < 0.0001 (****), with ns indicating no significant difference.

## 3. Results

### 3.1. PPS alleviates bladder dysfunction in mice with CYP-induced IC/BPS

**Figure 1:**
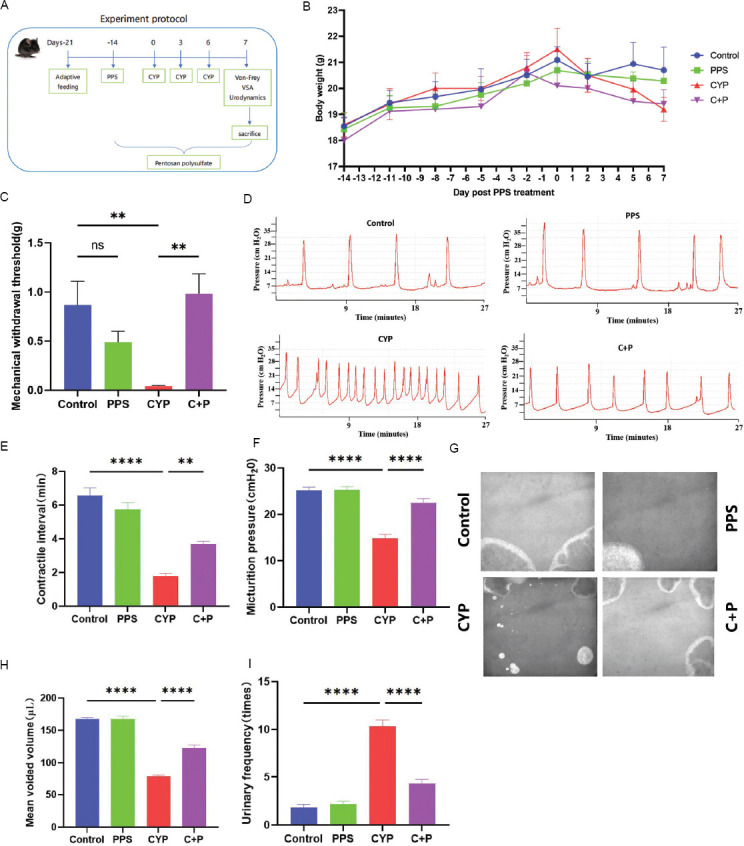
Pentosan polysulfate protects against bladder dysfunction in mice with CYP-induced interstitial cystitis/bladder pain syndrome. (A) Experimental workflow for assessing the protective effects of PPS on CYP-induced IC/BPS in mice. (B) Weight change curves of mice in four groups, n = 6. (C) Von Frey test results showed changes in mechanical pain thresholds in four groups of mice after PPS gavage and CYP modeling, n = 6. (D-F) Urodynamic plots and quantitative parameters of bladder function in four groups, n = 6. (G-I) Voiding stains and quantitative parameters of voiding behavior in four groups, n = 6. Note: Results are presented as mean ± standard error of the mean, **p < 0.01, ****p < 0.0001, analyzed by one-way analysis of variance. Abbreviations: C + P: Cyclophosphamide + pentosan polysulfate (CYP + PPS); CYP: Cyclophosphamide; IC/BPS: Interstitial cystitis/bladder pain syndrome; ns: Not significant; PPS: Pentosan polysulfate.

In our study, we evaluated protective effects of PPS on CYP-induced IC/BPS in mice. PPS was administered for 3 weeks, followed by CYP injections (50 mg/kg) on three occasions during the 4^th^ week, with continuous PPS gavage (Figure 1A). Body weight monitoring across four groups showed no significant changes, indicating no weight impact from PPS or CYP treatments (Figure 1B). The mechanical pain threshold was lower in the CYP group and higher in the PPS-treated CYP + PPS group, suggesting that PPS reduced CYP-induced pain (*p* < 0.001) (Figure 1C). Urodynamic tests (Figure 1D) showed that the CYP group had shorter voiding intervals and lower voiding pressure than the control (*p* < 0.0001) (Figure 1E), while the CYP + PPS group exhibited improvements, indicating PPS exerts beneficial effects on bladder function (*p* < 0.01) (Figure 1F). Voiding stain (Figure 1G) analysis also confirmed PPS increased voiding volume and decreased frequency in the CYP + PPS group compared to the CYP group (*p* < 0.0001) (Figure 1H and I), further supporting PPS’s role in mitigating bladder dysfunction similar to IC/BPS.

### 3.2. PPS mitigates CYP-induced bladder inflammatory infiltration and barrier damage in mice with IC/BPS

**Figure 2:**
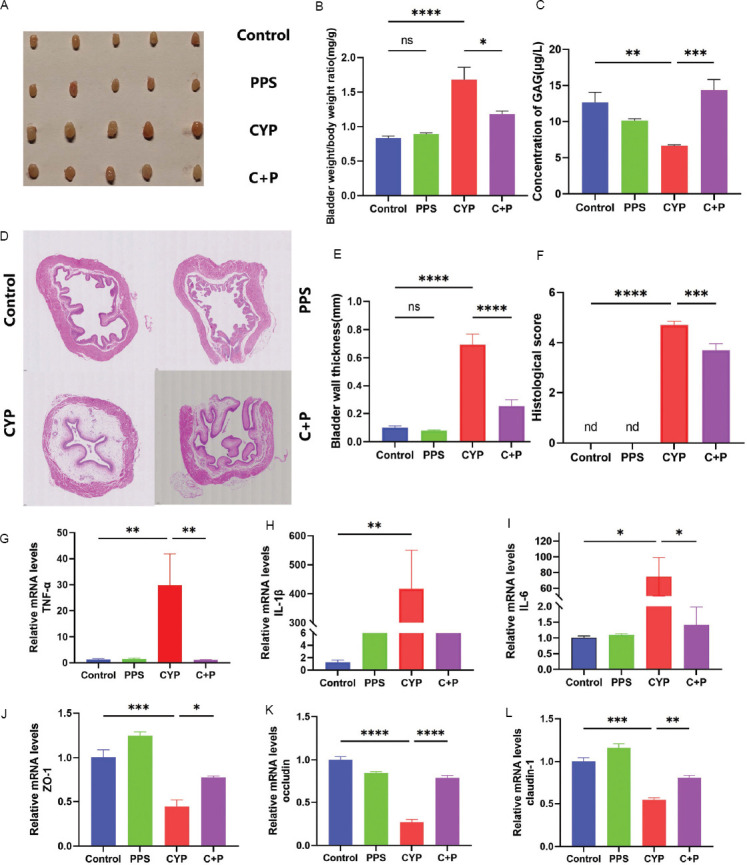
Pentosan polysulfate protects against bladder inflammation and barrier damage in CYP-induced interstitial cystitis/bladder pain syndrome mice. (A) Gross appearance of bladders from four groups of mice. (B) Bladder-to-body weight ratios in four groups of mice, n = 6. (C) GAG concentrations in bladder tissues, detected by ELISA, n = 6. (D-F) Hematoxylin and eosin staining, quantitative scores, and pathological damage scores of bladder tissues, scale bar: 50 μm, magnification: 20×, n = 5. (G-I) Expression levels of inflammatory cytokines TNF-α, IL-1β, and IL-6 in bladder tissues, n = 6. (J-L) mRNA expression levels of tight junction proteins ZO-1, occludin, and claudin-1 in bladder tissues, n = 6. Note: Results are presented as mean ± SEM, *p < 0.05, **p < 0.01, ***p < 0.001, ****p < 0.0001, analyzed by one-way analysis of variance. Abbreviations: C + P: Cyclophosphamide + pentosan polysulfate (CYP + PPS); CYP: Cyclophosphamide; GAG: Glycosaminoglycan; IC/BPS: Interstitial cystitis/bladder pain syndrome; ns: Not significant; PPS: Pentosan polysulfate.

In our study, mice in the CYP group presented significant edema, which was ameliorated in the PPS-treated CYP+PPS group (Figure 2A). The bladder-to-body weight ratio confirmed this observation, with an increase in the CYP group and a decrease in the CYP + PPS group (*p* < 0.0001 and *p* < 0.05, respectively) (Figure 2B). ELISA revealed lower GAG levels in the CYP group and higher levels in the CYP + PPS group (*p* < 0.01 and *p* < 0.001, respectively) (Figure 2C), indicating PPS has protective effect on bladder tissue.

The results of HE staining revealed that the bladder mucosa of mice in the control group was intact and devoid of interstitial hyperemia or edema. In contrast, the CYP group exhibited bladder epithelial mucosa shedding and defects, accompanied by submucosal lamina propria edema. The bladder epithelium of mice in the CYP + PPS group demonstrated better integrity and lesser lamina propria edema (Figure 2D). Compared to the control group, submucosal lamina propria in the CYP group showed a significant increase in submucosal spacing (*p* < 0.0001) (Figure 2E) and a corresponding elevation in HE scores (*p* < 0.0001) (Figure 2F). Conversely, the CYP + PPS group exhibited smaller submucosal spacing (*p* < 0.0001) (Figure 2E) and lower HE scores (*p* < 0.001) (Figure 2F).

In comparison with the control group, the mRNA levels of tumor necrosis factor-alpha (TNF-α) (Figure 2G), interleukin-1β (IL-1β) (Figure 2H), and IL-6 (Figure 2I) were significantly upregulated (*p* < 0.01, *p* < 0.05, and *p* < 0.01, respectively) in the CYP group. However, these levels were markedly downregulated in the CYP + PPS group (Figure 2G-I). Furthermore, the mRNA levels of tight junction proteins ZO-1 (Figure 2J), occludin (Figure 2K), and claudin-1 (Figure 2L) were significantly decreased in the CYP group but increased in the CYP + PPS group (*p* < 0.001, *p* < 0.0001, and *p* < 0.001, respectively). Collectively, these findings suggest that PPS alleviates CYP-induced inflammatory infiltration and barrier damage in the bladder of mouse IC/BPS models.

### 3.3. PPS intervention alters the gut microbiota composition in mice with CYP-induced IC/BPS

**Figure S2:**
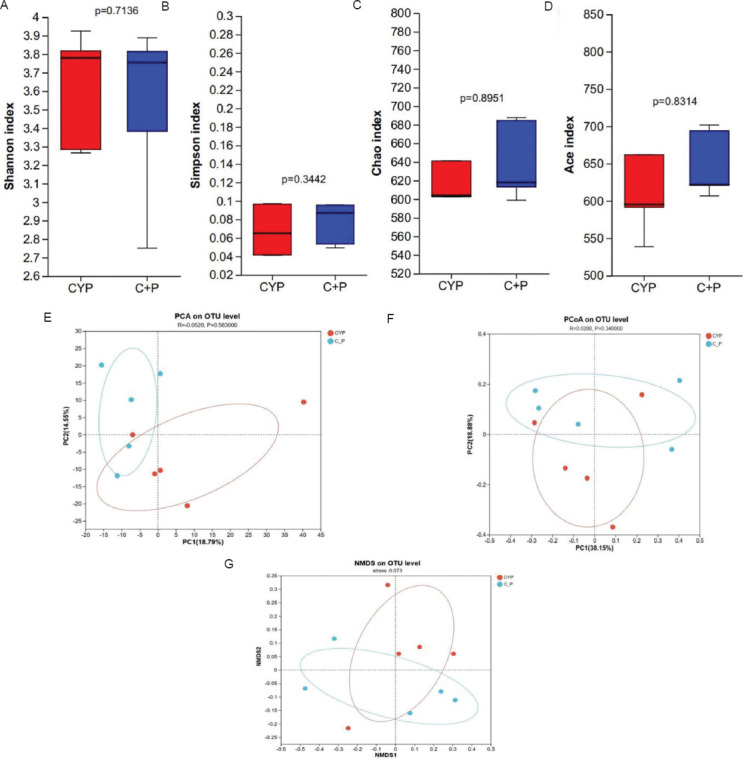
Analysis of microbial diversity through 16S rDNA sequencing. Microbial α-diversity was assessed using (A) Shannon index, (B) Simpson index, (C) Chao1 index, and (D) Ace index. Microbial β-diversity was evaluated through (E) principal component analysis (PCA), (F) principal coordinates analysis (PCoA), and (G) non-metric multidimensional scaling (NMDS). Abbreviations: C + P: Cyclophosphamide + Pentosan polysulfate; CYP: Cyclophosphamide; OTU: Operational taxonomic unit.

To examine the impact of PPS intervention on the gut microbiota of mice with CYP-induced IC/BPS, cecal contents were collected from the mice for 16s rDNA sequencing. An analysis of the gut microbiota diversity in mice was conducted. α-diversity analysis showed no significant differences in α-diversity indices, such as Shannon, Simpson, Chao1, and Ace, between the CYP group and the CYP+PPS group (Figure S2A-D). Similarly, β-diversity analysis revealed no significant differences in β-diversity indices in the principal component analysis (PCA), PCoA, and non-metric multidimensional scaling, between the two groups (Figure S2E and F). Since the IC/BPS model induced by intraperitoneal injection of CYP essentially represents chemical cystitis,^19^ and therefore is an extraintestinal disease model, there were no significant changes in the diversity of the gut microbiota.

**Figure 3:**
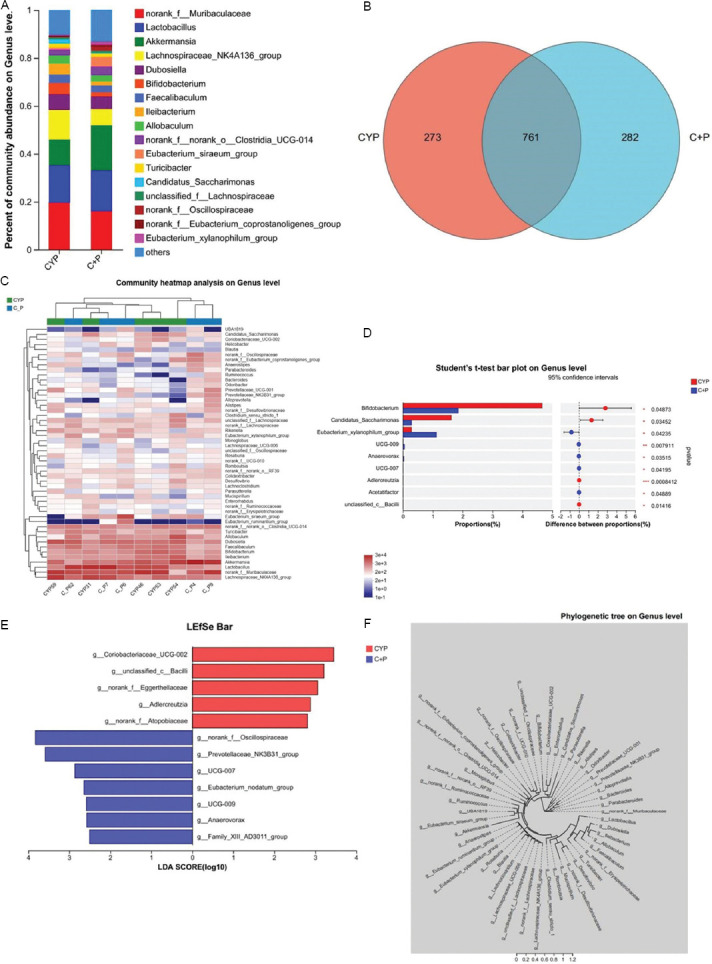
16S rDNA sequencing revealed Pentosan polysulfate-mediated alterations in gut microbiota composition in CYP-treated mice. (A) Bar plot showing differences in gut microbiota composition at the genus level between CYP and C + P groups, n = 5. (B) Venn diagram of gut microbiota composition between the CYP group and the C + P group. (C) Heatmap of differences in gut microbiota composition at the genus level between CYP and C + P groups, n = 5. (D) Differences in gut microbiota composition between CYP and C + P groups at the genus level, n = 5. (E) LEfSe analysis for identifying differentially abundant taxa. (F) Cladogram representation of taxonomic differences, n = 5 for both CYP and C + P groups. Abbreviations: C + P: Cyclophosphamide + Pentosan polysulfate (CYP + PPS); CYP: Cyclophosphamide; PPS: Pentosan polysulfate.

However, an analysis of the composition of the gut microbiota revealed that, at the genus level, compared with the CYP group, the CYP + PPS group showed decreased abundances of *Bifidobacterium*, *Ileibacterium*, *Allobaculum*, *Dubosiella*, *Faecalibaculum*, and *Lachnospiraceae NK4A136* groups, while increased abundances of *Eubacterium xylanophilum* group, *Akkermansia*, and *Lactobacillus* (Figure 3A). Venn diagram results showed that there were 761 overlapping species in the gut microbiota composition between the CYP group and the CYP+PPS group (Figure 3B). A heatmap of the species composition at the genus level in the two groups demonstrated that the abundances of *the E. xylanophilum* group *and Eubacterium nodatum* group were significantly increased (Figure 3C). Subsequent differential testing of the gut microbiota between the two groups showed that, compared with the CYP group, the CYP+PPS group had significantly increased abundances of *E. xylanophilum*, *Ruminococcus_UCG-009*, *Anaerovorax*, *Ruminococcus UCG-007*, and *Acetatifactor*, while *Bifidobacterium*, *Adlercreutzia*, *Candidatus* *Saccharimonas*, and *unclassified c Bacili* were significantly decreased (Figure 3D). LEfSe analysis (Figure 3E) and cladogram (Figure 3F) confirmed that the CYP group and the CYP+PPS group had distinct gut microbiota compositions, with *norank_f_Oscillospiraceae*, *Prevotellaceae_NK3B31_*group, *Ruminococcus UCG-007*, and *E. nodatum* group highly enriched in the CYP+PPS group. The results indicate that there are significant differences in the composition of the gut microbiota between the CYP group and the CYP+PPS group, and PPS significantly alters the gut microbiota composition of mice with CYP-induced IC/BPS.

### 3.4. PPS changes the gut microbiota metabolites in CYP-induced IC/BPS mice

**Figure 4:**
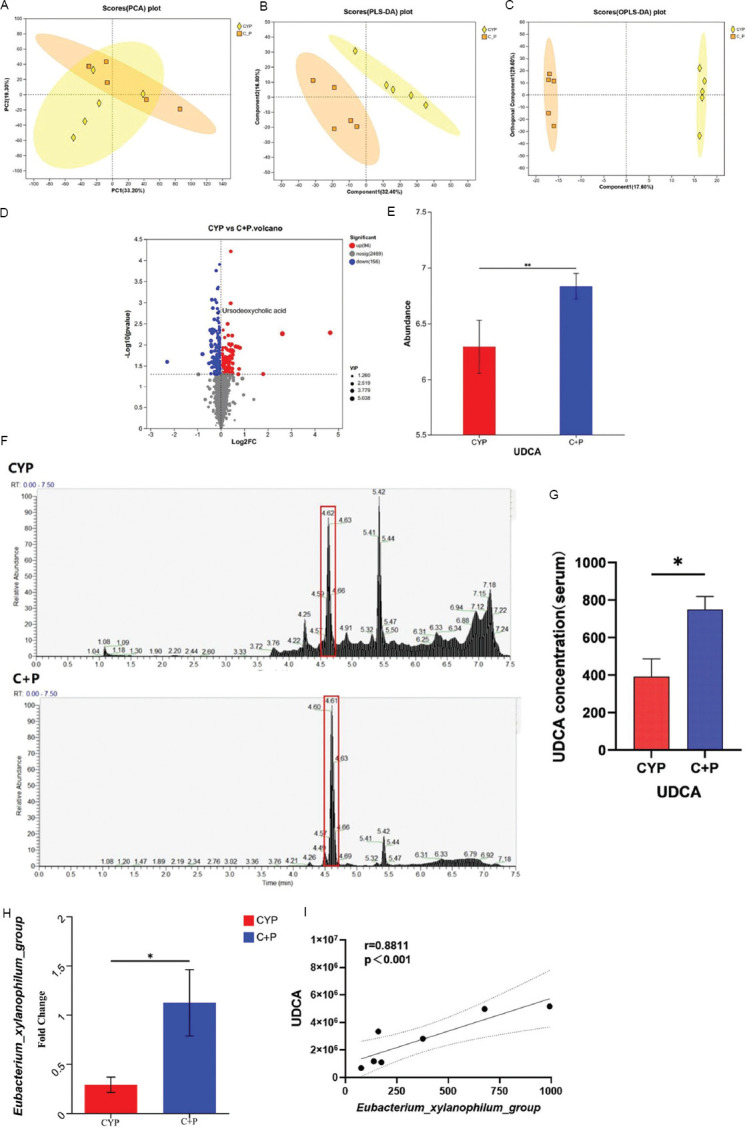
Pentosan polysulfate intervention alters the composition of intestinal and serum metabolites in CYP-treated mice. (A-C) Principal component analysis (PCA), partial least squares discriminant analysis (PLS-DA), and OPLS-DA of untargeted metabolomic data. (D) Volcano plot showing differential metabolites between CYP and C + P groups. (E) UDCA concentrations in cecal contents of two groups. (F-G) LC-MS targeted the detection of UDCA concentrations in the serum of two groups of mice. (H) Relative abundance of *Eubacterium xylanophilum* group in two groups, n = 5. (I) Scatter plot analysis of the correlation between UDCA concentrations in cecal contents and relative abundance of *E. xylanophilum* group, n = 7. Note: Statistical significance determined at *p < 0.05, **p < 0.01, analyzed by Student’s *t*-test. Abbreviations: C + P: Cyclophosphamide + Pentosan polysulfate (CYP + PPS); CYP: Cyclophosphamide; LC-MS: Liquid chromatography–mass spectrometry; PPS: Pentosan polysulfate; UDCA: Ursodeoxycholic acid.

To look into the changes in gut microbiota metabolites in mice following oral PPS administration, this study employed non-targeted metabolomics combined with LC/MS mass spectrometry to detect alterations in gut microbiota metabolites in cecal contents and serum of mice. PCA, partial least squares discriminant analysis (PLS-DA), and OPLS-DA results indicated that the CYP group and the CYP + PPS group had unique gut microbiota metabolite profiles with significant differences found between the two groups (Figure 4A-C). A volcano plot was generated based on a threshold of *p* < 0.05 and VIP ≥1. Among the 250 differential metabolites, 156 in cecal contents were found to be increased, and 94 were decreased in the CYP+PPS group compared to the CYP group (Figure 4D). Among the 156 increased metabolites, UDCA was selected and found to be significantly elevated (*p* < 0.01) in the CYP+PPS group (Figure 4E).

To verify the systemic changes of this secondary bile acid in mice, LC/MS mass spectrometry was used to target UDCA in mouse serum. Results showed that UDCA levels were significantly increased (*p* < 0.05) in the serum of mice in the CYP + PPS group (Figure 4F and G). UDCA may be a product of PPS metabolism through the gut microbiota and enter the bloodstream, reaching the bladder to ease CYP-induced IC/BPS. Previous literature has reported that *Eubacterium aerofaciens*, isolated from healthy human feces, can interact with *Bacteroides fragilis* or *Escherichia coli in vitro* to convert chenodeoxycholic acid into UDCA.^20^ While xylan, the precursor of PPS, cannot be digested and absorbed by humans, gut microbiota enzymes can degrade it into prebiotic metabolites that regulate the microbiome and impact human health.^21^

Therefore, this study looked at the relative abundance of *Eubacterium* and found that the abundance of *E. xylanophilum group* in cecal contents of mice in the CYP + PPS group was significantly increased compared to the CYP group (*p* < 0.05) (Figure 4H). Correlation analysis revealed a positive correlation between UDCA content and the abundance of the *E. xylanophilum* group (*p* < 0.001, r = 0.8811) (Figure 4I). These results suggest that, upon oral PPS administration, the gut microbiota in mice produces UDCA and releases it into the bloodstream. The *E. xylanophilum* group, which metabolizes xylan, may play a part in PPS catabolism, producing UDCA that alleviates CYP-induced IC/BPS.

### 3.5. FMT from CYP + PPS mice alleviates CYP-induced IC/BPS in mice

**Figure 5:**
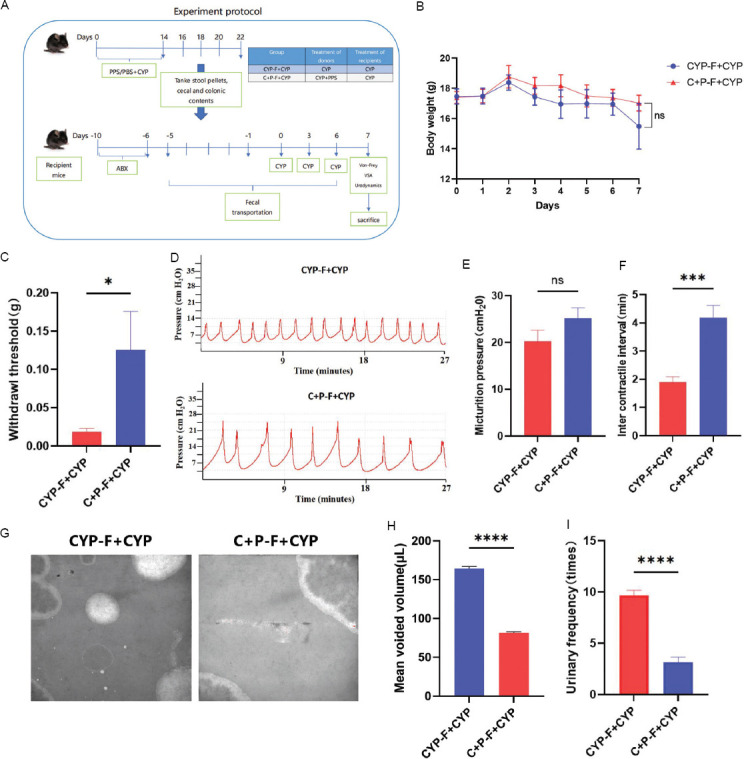
FMT from CYP + Pentosan polysulfate mice alleviates CYP-induced bladder dysfunction. (A) Experimental workflow for assessing the effects of FMT from C + P mice on CYP-induced IC/BPS in mice. (B) Weight change curves of two groups of mice, n = 6. (C) Von Frey test results showed changes in mechanical pain thresholds in two groups, n = 6. (D-F) Urodynamic plots and quantitative parameters of bladder function in two groups, n = 6. (G-I) Voiding stains and quantitative parameters of voiding behavior in two groups, n = 6. Note: Results are presented as mean ± SEM, **p < 0.05, ***p < 0.001, ****p < 0.0001, analyzed by Student’s *t*-test. Abbreviations: C + P: Cyclophosphamide + Pentosan polysulfate (CYP + PPS); CYP: Cyclophosphamide; FMT: Fecal microbiota transplantation; IC/BPS: Interstitial cystitis/bladder pain syndrome; ns: Not significant; PPS: Pentosan polysulfate.

**Figure 6:**
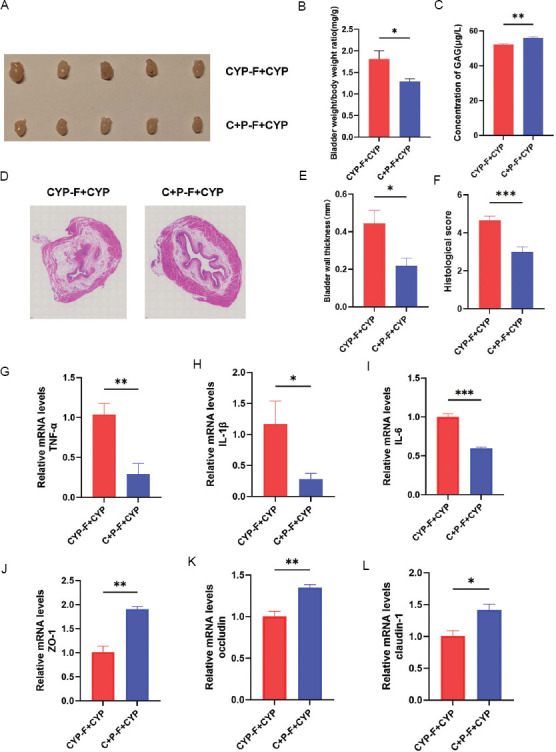
FMT from CYP + Pentosan polysulfate mice alleviates CYP-induced bladder inflammation and barrier damage. (A) Gross appearance of bladders from two groups. (B) Bladder-to-body weight ratios in two groups, n = 6. (C) GAG concentrations in bladder tissues detected by ELISA, n = 6. (D-F) HE staining, quantitative scores, and pathological damage scores of bladder tissues, scale bar: 50 μM, magnification: 20×, n = 5. (G-I) Expression levels of inflammatory cytokines TNF-α, IL-1β, and IL-6 in bladder tissues, n = 6. (J-I) mRNA expression levels of tight junction proteins ZO-1, occludin, and claudin-1 in bladder tissues, n = 6. Note: Results are presented as mean ± SEM, *p < 0.05, ***p < 0.01, ***p < 0.001, analyzed by Student’s *t*-test. Abbreviations: C + P: Cyclophosphamide + Pentosan polysulfate (CYP + PPS); CYP: Cyclophosphamide; FMT: Fecal microbiota transplantation; GAG: Glycosaminoglycan; IC/BPS: Interstitial cystitis/bladder pain syndrome; ns: Not significant; PPS: Pentosan polysulfate.

To determine if the gut microbiota mediates PPS’s alleviation of CYP-induced IC/BPS in mice, mice were treated with a broad-spectrum antibiotic cocktail for 5 days to deplete their gut microbiota. Following this, FMT from both CYP and CYP+PPS groups was performed in the antibiotic-treated mice, followed by CYP-induced IC/BPS (Figure 5A). Monitoring in the two groups showed no significant changes in body weight, indicating no impact results from CYP and CYP+PPS FMT treatments (Figure 5B). Mice receiving FMT from the CYP+PPS group showed a higher mechanical pain threshold (*p* < 0.05) (Figure 5C), increased voiding interval (*p* < 0.001) (Figure 5D and F), a significant improvement in voiding volume and frequency (*p* < 0.0001) (Figure 5G-I) compared to those receiving FMT from the CYP group, although average voiding pressure exhibited no significant changes (Figure 5E). Macroscopic analysis of bladders from CYP-modeled mice treated with CYP + PPS group FMT showed reduced congestion and edema (Figure 6A). The bladder-to-body weight ratio and GAG content were decreased and increased, respectively, in the CYP + PPS – FMT + CYP group (*p* < 0.05 and *p* < 0.01, respectively) (Figure 6B and C).

Histological evaluation (HE staining) indicated improved bladder epithelial integrity and reduced edema in the CYP + PPS – FMT + CYP group compared to the CYP – FMT + CYP group (Figure 6D). In addition, submucosal spacing was significantly reduced (*p* < 0.05) (Figure 6E), and pathological damage scores were lower (*p* < 0.001) (Figure 6F) in the CYP + PPS – FMT + CYP group. qPCR revealed increased mRNA levels of inflammatory cytokines TNF-α, IL-1β, and IL-6 and decreased levels of tight junction proteins ZO-1, occludin, and claudin-1 in the CYP + PPS – FMT + CYP group (*p* < 0.01, *p* < 0.05, and *p* < 0.001, respectively) (Figure 6G-L). These results indicate that PPS mitigates CYP-induced bladder inflammation, dysfunction, and barrier damage through the gut microbiota.

### 3.6. UDCA eases lipopolysaccharide-induced inflammatory infiltration and barrier damage in SV-HUC-1 cells by activating TGR5

**Figure S3:**
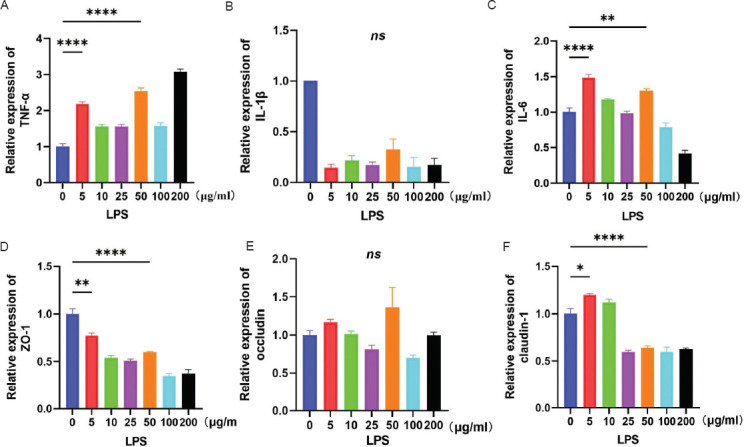
Successful establishment of an IC/BPS cell model in SV-HUC-1 cells induced by lipopolysaccharide. (A–C) Changes in mRNA levels of inflammatory cytokines TNF-α, IL-1β, and IL-6 after incubation with different concentrations of LPS for 24 h in SV-HUC-1 cells, n = 3. (D–F) Changes in mRNA levels of bladder epithelial barrier tight junction proteins ZO-1, occludin, and claudin-1 after incubation with different concentrations of LPS for 24 h in SV-HUC-1 cells, n = 3. Note: Results are presented as mean ± SEM, with statistical significance indicated by *p < 0.05, **p < 0.01, ****p < 0.0001 (one-way analysis of variance). Abbreviations: LPS: Lipopolysaccharide; ns: Not significant.

Metabolomic sequencing has revealed that PPS alleviates CYP-induced IC/BPS in mice, potentially through the intestinal microbial metabolite UDCA. In this study, SV-HUC-1 cells were selected to investigate the protective mechanism of UDCA. First, the appropriate LPS concentration to induce an IC/BPS model in SV-HUC-1 cells was explored. As shown in Figure S3, incubation of SV-HUC-1 cells with LPS at 5 μg/mL or 50 μg/mL for 24 h resulted in increased levels of inflammatory cytokines TNF-α (Figure S3A) and IL-6 (Figure S3C), and decreased levels of barrier tight junction proteins ZO-1 (Figure S3D) and claudin-1 (Figure S3F).

**Figure S4:**
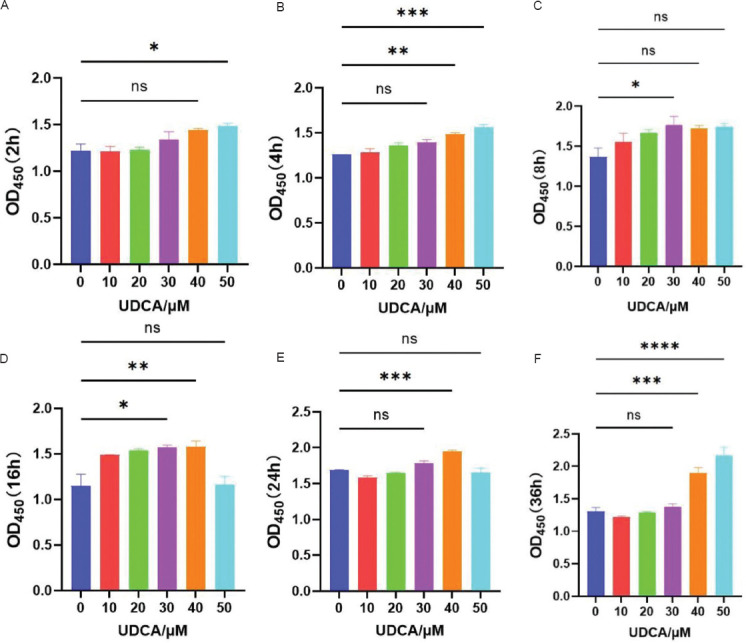
The toxic effects of ursodeoxycholic acid on SV-HUC-1 cells at different concentrations using CCK8. Optical density (OD) values of SV-HUC-1 cells treated with UDCA at concentrations of 10 μM, 20 μM, 30 μM, 40 μM, and 50μM for durations of (A) 2 h, (B) 4 h, (C) 8 h, (D) 16 h, (E) 24 h, and (F) 32 h. Note: Results are presented as mean ± SE, with statistical significance indicated by *p < 0.05, **p < 0.01, ***p < 0.001, ****p < 0.0001 (one-way analysis of variance). Abbreviations: ns: Not significant; UDCA: Ursodeoxycholic acid.

Premised on these results, an LPS concentration of 50 μg/mL was chosen to induce IC/BPS in SV-HUC-1 cells. Next, the toxic effects of UDCA on SV-HUC-1 cells were investigated to determine the appropriate intervention concentration. Five concentrations of UDCA (10, 20, 30, 40, and 50 μM) were used, and SV-HUC-1 cells were treated for 2, 4, 8, 16, and 32 h. Cell viability was assessed using CCK8 at each time point. As shown in Figure S4, UDCA had no toxic effects on SV-HUC-1 cells within the concentration range of 10 – 50 μM and the treatment duration of 2 – 36 h. Therefore, a concentration of 30 μM UDCA was chosen for subsequent experiments. SV-HUC-1 cells were pretreated with 30 μM UDCA for 2 h before exposure to 50 μg/mL LPS.

**Figure 7:**
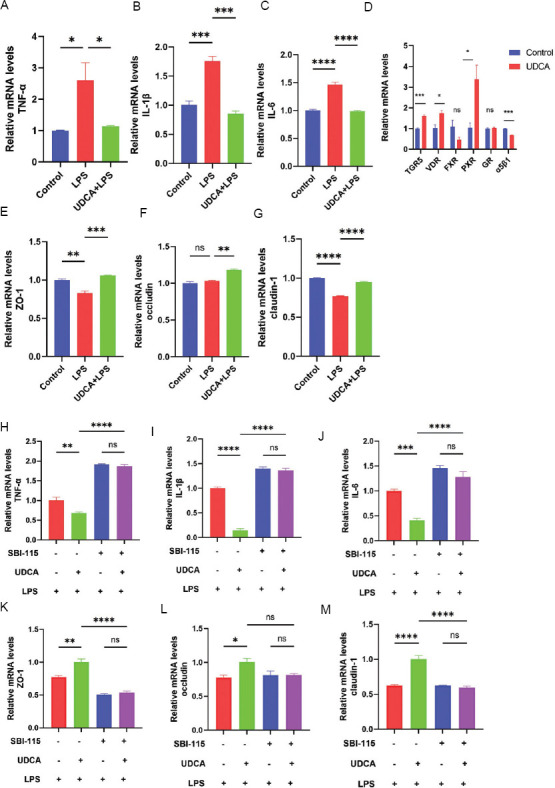
Inhibition of TGR5 by SBI-115 confirmed that UDCA alleviated lipopolysaccharide-induced inflammation and barrier damage in SV-HUC-1 cells by activating TGR5. (A-C) mRNA expression levels of inflammatory cytokines TNF-α, IL-1β, and IL-6 after SV-HUC-1 cells were pretreated with UDCA for 2 h and incubated with LPS for 24 h. (D) mRNA expression levels of TGR5, VDR, FXR, PXR, GR, and α5β1 after UDCA incubation for 24 h. (E-G) mRNA expression levels of tight junction proteins ZO-1, occludin, and claudin-1 after SV-HUC-1 cells were pretreated with UDCA for 2 h and incubated with LPS for 24 h. (H-J) mRNA expression levels of inflammatory cytokines TNF-α, IL-1β, and IL-6 and (K-M) mRNA expression levels of tight junction proteins ZO-1, occludin, and claudin-1 after SV-HUC-1 cells were pretreated with SBI-115 for 2 h and incubated with LPS for 24 h. Note: Results are presented as mean ± SEM, *p < 0.05, ***p < 0.01, ***p < 0.001, ****p < 0.0001, analyzed by one-way analysis of variance (A-C, E-M) or Student’s *t*-test (D). Abbreviations: LPS: Lipopolysaccharide; ns: Not significant; UDCA: Ursodeoxycholic acid.

qPCR revealed that UDCA pre-treatment significantly reduced mRNA levels of inflammatory cytokines TNF-α (*p* < 0.05) (Figure 7A), IL-1β (*p* < 0.001) (Figure 7B), and IL-6 (*p* < 0.0001) (Figure 7C) and increased mRNA levels of tight junction proteins ZO-1 (*p* < 0.001) (Figure 7E), occludin (*p* < 0.01) (Figure 7F), and claudin-1 (*p* < 0.0001) (Figure 7G). These results suggest that UDCA lessened LPS-induced inflammatory infiltration and barrier damage in SV-HUC-1 cells. In addition, UDCA treatment increased mRNA levels of bile acid receptors TGR5 (*p* < 0.001), VDR (*p* < 0.05), and PXR (*p* < 0.05), with TGR5 selected for further mechanistic investigation (Figure 7D).

**Figure 8:**
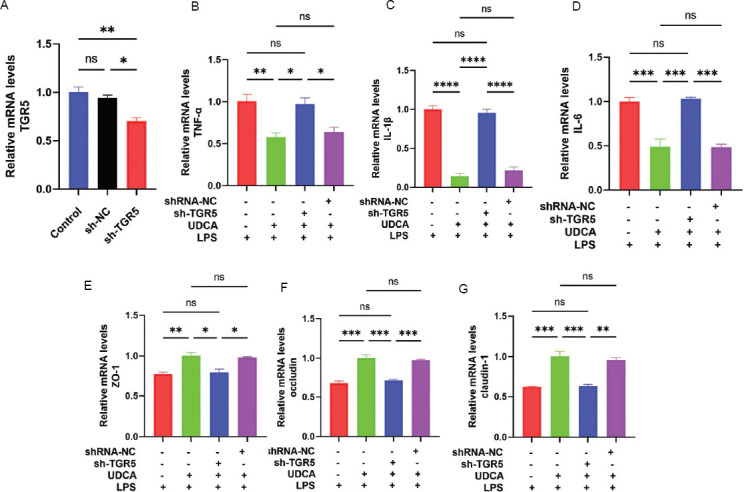
shRNA-mediated knockdown confirmed that UDCA alleviated lipopolysaccharide-induced inflammation and barrier damage in SV-HUC-1 cells by activating TGR5. (A) qPCR validation of shRNA-mediated knockdown of TGR5. (B-D) mRNA expression levels of inflammatory cytokines TNF-α, IL-1β, and IL-6. (E-G) mRNA expression levels of tight junction proteins ZO-1, occludin, and claudin-1 after constructing stable SV-HUC-1 cell lines with knockdown of TGR5 and pretreating them with UDCA for 2 h, followed by LPS incubation for 24 h. Results are presented as mean ± SEM, *p < 0.05, **p < 0.01, ***p < 0.001, ****p < 0.0001, analyzed by one-way analysis of variance. Abbreviations: LPS: Lipopolysaccharide; ns: Not significant; qPCR: Quantitative polymerase chain reaction; UDCA: Ursodeoxycholic acid; SEM: Scanning electron microscopy.

To verify whether UDCA alleviates LPS-induced IC/BPS by activating TGR5, a small molecule compound SBI-115 was used to inhibit TGR5 activity. As shown in Figure 7, compared to the LPS group, in the UDCA+LPS group, mRNA levels of inflammatory cytokines (TNF-α, IL-1β, IL-6) were significantly increased (Figure 7H-J) and mRNA levels of tight junction proteins (ZO-1, occludin, claudin-1) decreased (Figure 7K-M). However, treatment with SBI-115 inhibited the protective effect of UDCA on LPS-induced IC/BPS in cells. Furthermore, SV-HUC-1 cells with TGR5 knockdown using shRNA were constructed to explore the mechanism of UDCA alleviating IC/BPS. Figure 8A shows that a stable cell line of SV-HUC-1 with TGR5 knocked down was successfully established and verified using qPCR. Compared to the LPS group, the UDCA + LPS group showed significantly increased mRNA levels of inflammatory cytokines (TNF-α, IL-1β, IL-6) (Figure 8B-D) and decreased mRNA levels of tight junction proteins (ZO-1, occludin, claudin-1) (Figure 8E-G). Nonetheless, TGR5 knockdown significantly inhibited the protective effect of UDCA on LPS-induced IC/BPS in cells (Figure 8B-G). In summary, this study demonstrated that UDCA alleviates LPS-induced inflammatory infiltration and barrier damage in SV-HUC-1 cells through TGR5 activation.

## 4. Discussion

IC/BPS is characterized primarily by chronic pelvic pain associated with the bladder, accompanied by persistent lower urinary tract symptoms lasting more than 6 weeks, without infection or a specific etiology.^35^ The etiopathology of IC/BPS is complex, involving multiple regulatory mechanisms. However, growing experimental and clinical evidence suggests that functional impairment of the urothelial barrier represents the final stage of IC/BPS.^36^ PPS has been internationally approved as a therapeutic drug for IC/BPS. Despite this, its mechanism of action is not fully understood, and the role of PPS in the gut microbiota remains unexplored. Our research results indicate that PPS can significantly alleviate the inflammation of the bladder urothelium in IC/BPS and promote barrier function repair by modulating the gut microbiota, particularly the abundance of the *E. xylanophilum* group. PPS has been shown to work as an analog of GAGS in the urothelium, thereby positively contributing to the repair of bladder barrier function.^37^ In addition, we found that the *E. xylanophilum* group is involved in the biosynthesis and metabolism of secondary bile acids, thereby increasing the abundance of UDCA in the circulating pool. This UDCA enters the systemic blood circulation and ultimately reaches the bladder urothelium to activate TGR5, helping alleviate inflammation and barrier disruption in IC/BPS. Increasing studies suggest that the interplay between bladder mucosal dysfunction and gut microbiota disruption is one of the key triggering factors in the development of IC/BPS due to the gut-bladder axis.^8^ There is a positive correlation between the risk of IC/BPS and eight gut microbiota taxa, including the genera *Bacteroides*, *Haemophilus*, *Veillonella*, *Coprococcus 1*, *Butyricimonas*, the family *Bacteroidaceae*, the family *Christensenellaceae*, and the order *Lactobacillales*.^38^ Prebiotics are controllable factors that can normalize intestinal permeability, balance immune responses, and alleviate intestinal and extraintestinal symptoms by modulating the composition of the gut microbiota.^39^ Our results showed that PPS, as a prebiotic, can restore the gut microbiota composition in murine IC/BPS model, thus increasing the abundance of the *E. xylanophilum* group, which is positively correlated with higher levels of the protective metabolite UDCA.

Furthermore, we observed that transferring the gut microbiota from PPS-treated mice to IC/BPS model recipient mice yielded transferable protective effects. These results indicate that gut microbiota plays a crucial role in the efficacy of PPS in treating IC/BPS. Although there have been reports of gut microbiota influencing the development of IC/BPS, the potential determinants at the cellular or molecular levels and the affected host pathways have not been fully characterized. More and more evidence suggests that the gut functions as an “endocrine organ,” with the produced metabolites working as key signaling molecules in microbial communities and host-microbial crosstalk.^40^ Bile acid conversion is a coordinated process between the host and gut microbiota. The gut microbiota chemically modifies primary bile acids to produce secondary bile acids, the modification playing a key role in the regulation of the immune system. These secondary bile acids can either enhance or inhibit inflammatory responses and help maintain metabolic homeostasis by activating the farnesoid X receptor and various other nuclear receptors.^41^ The composition of the gut microbiota affects host’s composition of bile acid pools.^42^ Our results suggest that PPS works along an intraluminal metabolic pathway that increases the abundance of the *E. xylanophilum* group, which is involved in the synthesis of the secondary bile acid UDCA. UDCA can bind to the bile acid membrane receptor TGR5, increase sphingosine-1-phosphate (S1P) levels, and enhance the ability of S1PR2 to bind to S1P. These processes alleviate excessive inflammatory responses triggered by accumulated NF-κB and c-Jun N-terminal kinases by downregulating serine/threonine kinase expression and inhibiting inflammatory cytokines.^43^ Moreover, we observed that blocking TGR5 with the inhibitor SBI-115 and sh-TGR5 significantly inhibited the protective effects of UDCA, that is, alleviating inflammation and restoring barrier function in IC/BPS. At the same time, it must be noted that the notion of how the *E. xylanophilum* group metabolizes PPS to participate in UDCA biosynthesis is primarily based on literature and conjecture. Moreover, the protective effect of PPS is not solely attributed to UDCA, but a wide spectrum of protective metabolites may well mediate the action.

The discovery that PPS modulates the gut microbiota to alleviate IC/BPS symptoms provides a novel therapeutic pathway. This approach could be particularly beneficial in managing IC/BPS by targeting the gut microbiota, which is being increasingly recognized for its role in various diseases. PPS as a prebiotic potentially offers a non-invasive treatment option that could complement existing therapies. While our study provides valuable insights into the role of PPS in IC/BPS, it is subject to several limitations. The primary limitation is the lack of clinical data to support our findings. Future studies should include clinical trials to validate the efficacy of PPS in modulating the gut microbiota and alleviating IC/BPS symptoms. In addition, the long-term and potential side effects of PPS treatment in IC/BPS patients have yet to be determined, warranting further investigation. Furthermore, the interaction between PPS, the gut microbiota, and the urothelium is complicated and may vary with individuals. More research is needed to understand the heterogeneity in treatment response and to develop personalized treatment strategies based on individual microbiota profiles.

## 5. Conclusion

From the perspective of intestinal microecology, this study developed *in vivo* and *in vitro* models of IC/BPS by intraperitoneally injecting CYP to C57BL/6J mice and treating SV-HUC-1 cells with LPS. By using these models, we looked into the protective effects of PPS on the bladder during IC/BPS, and the relevant mechanisms. We, for the first time, demonstrated that PPS may act as a prebiotic by enhancing the metabolism of the xylan-degrading bacterium, *E. xylanophilum* group, thereby increasing the production of UDCA. The elevated UDCA activates the TGR5 receptor on bladder epithelial cells, improving bladder function of IC/BPS mice and alleviating bladder inflammation, infiltration, and barrier damage. These discoveries provide novel theoretical insights for current clinical treatments of IC/BPS. In addition, the results of this study laid an experimental foundation for refining the “gut-bladder axis” theory.

## Data Availability

All data generated or analyzed during this study are included in this article and the supplementary information files.
